# Selection of microbial biomarkers with genetic algorithm and principal component analysis

**DOI:** 10.1186/s12859-019-3001-4

**Published:** 2019-12-10

**Authors:** Ping Zhang, Nicholas P. West, Pin-Yen Chen, Mike W. C. Thang, Gareth Price, Allan W. Cripps, Amanda J. Cox

**Affiliations:** 1 Menzies Health Institute QLD, Griffith University, Gold Coast, Australia; 2 School of Medical Science, Griffith University, Gold Coast, Australia; 3 QFAB Bioinformatics, Institute for Molecular Bioscience, University of Queensland, Brisbane, Australia; 4 School of Medicine, Griffith University, Gold Coast, Australia

**Keywords:** PCA, Genetic algorithm, Obesity, Biomarker

## Abstract

**Background:**

Principal components analysis (PCA) is often used to find characteristic patterns associated with certain diseases by reducing variable numbers before a predictive model is built, particularly when some variables are correlated. Usually, the first two or three components from PCA are used to determine whether individuals can be clustered into two classification groups based on pre-determined criteria: control and disease group. However, a combination of other components may exist which better distinguish diseased individuals from healthy controls. Genetic algorithms (GAs) can be useful and efficient for searching the best combination of variables to build a prediction model. This study aimed to develop a prediction model that combines PCA and a genetic algorithm (GA) for identifying sets of bacterial species associated with obesity and metabolic syndrome (Mets).

**Results:**

The prediction models built using the combination of principal components (PCs) selected by GA were compared to the models built using the top PCs that explained the most variance in the sample and to models built with selected original variables. The advantages of combining PCA with GA were demonstrated.

**Conclusions:**

The proposed algorithm overcomes the limitation of PCA for data analysis. It offers a new way to build prediction models that may improve the prediction accuracy. The variables included in the PCs that were selected by GA can be combined with flexibility for potential clinical applications. The algorithm can be useful for many biological studies where high dimensional data are collected with highly correlated variables.

## Background

Association between the human gut microbiome and a diverse range of health issues has been reported in a number of studies [1, 2]. Knight and colleagues [3] reviewed the methodological approach in microbiome studies, including: experimental design, choice of molecular analysis technology, methods for data analysis, and the integration of multiple -omics data sets. Different methods for surveying microbial communities include 16S ribosomal RNA, and metagenomic and metatranscriptomic sequencing. Next-step data analyses are needed to search for overall patterns in microbiome variation. The association between obesity and the gut microbiome from the phylum level to the species level has been studied and various results have been reported [4–6].

Several well-known sequence data analysis pipelines for microbiota study have been published, for example Quantitative Insights into Microbial Ecology (QIIME) [7], MetaGenome Rapid Annotation using Subsystem Technology (MG-RAST) [8] and mothur [9]. These packages include the functions of sequence alignment, operational taxonomic unit (OTU) identification, taxonomy classification, and alpha and beta diversity calculation. They have been widely used for different biological and medical research purposes, such as associating gut microbiome diversity with diseases [10–13]. It is important to recognise that due to some possible pitfalls in sample processing, the abundance of specific bacterial species and overall community composition can be distorted, thus hampering the analysis and threatening the validity of the research findings [14]. In addition, a key limitation of using 16S rRNA gene analysis for genus and species level classification is that related bacterial species may be indistinguishable due to near identical 16S rRNA gene sequences [15]. The potential for different data analysis approaches to produce different outcomes has also been recognised. Plummer et al. [15] compared three pipelines commonly used for 16S rRNA gene analysis: QIIME, MG-RAST and mothur. Favourably, their results showed that the three pipelines produced comparable results for analysis of faecal samples, in terms of alpha diversity and usability. Although a difference was observed between the pipelines in terms of taxonomic classification of genera from the Enterobacteriaceae family, the three pipelines detected the same phylum in similar abundances. D’Argenio et al. [16] also compared QIIME and MG-RAST, and observed a statistically significant difference between these two bioinformatics pipelines with regards to beta diversity measures.

Despite the effort from researchers to develop high quality analytical pipelines, it is recognised that the complexity and variability of the human microbiome can be sensitive to various environmental factors [17]. Improvement of analytical pipelines has been complicated by the limitation of available sample material and the relatively high cost of the sequence analysis necessary for microbiome profiling. As a result, most microbiome studies have used limited sample sizes, raising questions regarding the accuracy of their findings. In addition to efforts to improve the accuracy of OTU detection and taxonomic classification, especially at the genus and species levels, researchers have been studying ways to characterise diseases based on microbial composition. Rather than simply associating diseases and individual microbial features, such as a phylum or species, studies have started looking at defining microbial signatures for specific diseases. This includes the application of computational modelling and variable selection techniques. For example, Rivera-Pinto et al. [18] presented a greedy stepwise algorithm for selection of microbial signatures that preserves the principles of compositional data analysis. Sze and Schloss [19] performed a meta-analysis on associations between specific microbiome-based markers and obesity, concluding that although there was support for a relationship between human faecal microbial communities and obesity status, this association was relatively weak and its detection is confounded by large interpersonal variation and insufficient sample sizes. The same study also tested random forest models for classifying individuals as obese on the basis of microbiome composition and did not find obvious patterns that could separate the obese and healthy groups. Random forest models were also used by Peters et al. [20] to identify taxonomic signatures of obesity. These models were evaluated with Receiver Operator Characteristic (ROC) curves and the area under the curve (AUC) value produced by the optimal model, which included 49 OTUs, was 0.81. When the repeated cross-validation was performed, the AUC value decreased to 0.65. Other machine learning methods used for microbiome studies have been reviewed by Knights et al. [21].

With the potential for large numbers of microbial species to be identified in human faecal samples and the high correlation between many of the species detected, principal components analysis (PCA) is often used. Studies use PCA to find characteristic patterns associated with certain diseases by reducing variable numbers based on their correlation with a principal component (PC), before a predictive model is built. The first two or three principle components account for the greatest proportion of the variance in the dataset. Usually, these components are then used to determine whether individuals can be clustered into one of two classification groups, control or diseased, based on pre-determined criteria. However, we have asked the following questions: (i) Is it possible that the proportion of variance captured by the first two or three PCs is unrelated to the disease groups, and that the variance explained by other components is able to better distinguish disease individuals from healthy controls? (ii) Are there different groups of bacterial species associated with individual obesity?

With these questions in mind, we developed a prediction method that combines PCA and a genetic algorithm (GA) for microbial biomarkers identification. We applied this approach to faecal microbial data collected from our obesity study, to identify potential sets of bacterial species that may be associated with obesity with metabolic syndromes (MetS). The preliminary work has been presented in the 2018 IEEE International Conference on Bioinformatics and Biomedicine [22].

## Methods

### Principal components analysis

PCA is often used as a tool in exploratory data analysis for variable dimensionality reduction prior to building predictive models. It can be used to reduce a large number of predictor variables to a few PCs, particularly in datasets that are noisy or have strongly correlated explanatory variables. The PCs can then be used to build predictive models. The PCs are the linear combinations of the original variables that account for variance in the data. PCA can be performed using either eigenvalue decomposition of a data covariance matrix or singular value decomposition of a data matrix. The coefficients corresponding to each variable in the linear combinations indicate the relative weight of the variable in the component. The larger the absolute value of the coefficient, the more important the corresponding variable is in calculating the component. To make the coefficient value for each variable comparable, the data should be normalized to have the same unit of measurement before PCA is used.

### Genetic algorithms

GA is a search heuristic to find optimal solutions by mimicking Charles Darwin’s theory of natural evolution--fittest individuals are selected for reproduction for the next generation. In GA, the potential solutions compete and mate with each other to produce increasingly fitter individuals over multiple generations.

GAs can be useful and efficient when searching for the best combination of variables to achieve the best outcome (e.g. accuracy of prediction). GAs have been developed and applied for biomarker profile identification in a range of settings such as Alzheimer’s disease progression and breast cancer diagnosis [23–25]. The GAs have also been modified and improved to adapt to different computational environments and for different applications [26, 27]. Carter et al. [28] applied GA to their study to select vaginal microbiome features associated with bacterial vaginosis. However, the actual features were not reported, as authors explained that evaluation was needed from both microbial and clinical perspectives in the future.

In this study, GA will be used to find the best subset of principal components produced from a PCA using gut microbial species data.

### Proposed method

**Fig. 1:**
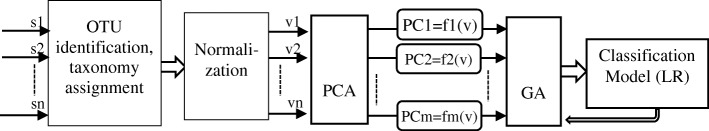
A diagram of proposed method. s1, s2...sn are the 16S rRNA sequences for this study (can be from other sequencing). v1 to vn are the abundance (normalized) of each species detected in each individual. m = number of PCs created by PCA, *n* = number of individuals included in the sample. PCA is used to produce PC scores for each individual, and GA is used to select the best subset of PCs to distinguish obesity from healthy cases

The method described here uses normalized OTU abundance with taxonomy assigned across the sample as the input for PCA. The OTUs can be identified by any of the sequence analysis pipelines mentioned above or other software packages, such as “DADA2” [29] in R (https://cran.r-project.org/). GA is then applied for selection of the set of components created from the PCA that best predict individuals as obese or healthy weight. The scores of selected PCs calculated for each individual are used as the input for building a classification model. ROC curve analysis is used to evaluate the classification models and is used as the fitness function for the GA. The method is shown diagrammatically in Fig. 1. In this research, logistic regression (LR) is used for building the classification models and more details about how to implement the GA can be found in reference [24].

## Experiments and results

In this study, faecal samples from 22 obese and 105 healthy-weight subjects were collected and sequenced using a 16S-based approach. The obese sample here was designed as those with body mass index (BMI) over 30 and with MetS [10]. The healthy-weight subjects included 39 recreational individuals and 66 athletes who were involved in rugby, football soccer, judo, rowing, triathlon or weightlifting. For sequencing analysis, paired-end reads were merged using the PEAR software (v0.9.6) [30]. Contaminant human reads were removed by mapping to the hg19 human genome using BWA software package (v0.7.12) [31] and the remaining reads were searched against the Greengenes 16S taxonomy database (GG v13.5) [32] using sequence analysis tool VSEARCH (v1.9.7) [33] to generate a single OTU raw count/abundance table for all 127 subjects. Amongst the 127 subjects 68,590 OTUs were identified (at all taxonomic levels), which mapped at the level of species to 163 observations, from Greengenes total reportable content of 3093 species. Species with low diversity across the cohort were filtered from future analysis, this was achieved by removing the species with zero abundance in 80% of both healthy and obese subjects. This excluded 126 species (77.3%) of the data leaving 37 species for further analysis. The abundance values of each of these species were normalized to the range of [0, 1] (highest abundance across the individuals as 1 and the lowest as 0) before applying the proposed method which combines PCA and GA for identifying obese from healthy subjects. The results were compared with those produced without GA and with those produced by using GA to select combinations of bacterial species for heathy and obese classification without PCA in the model.

### GA models to select combination of PCs for classification

For experiments, we performed PCA across three circumstances using: the whole dataset, obese only sample, and healthy weight only sample. This approach is based on the possibility that for different populations, the correlation between species might differ. The function “prcomp” from the “stats” package in R [34] was used to create the PCs and calculate the scores for each individual. These scores were then used to build the classification model with GA to select the best components for identifying obese from healthy subjects. The algorithm used by “prcomp” for creating the PCs can be found in reference [35]. Essentially, the PC calculation is performed by a singular value decomposition of the data matrix. If there are *n* observations with *p* variables, then the number of distinct PCs is *min(n,p)*.

GA was completed with the fitness function of the cross-validated AUC value created from the logistic regression model. More explanation about AUC can be found in Johnson et al. [24]. Constraints for GA were set to include 1 to 6 PCs in the classification model. Ten-times repeated five-fold cross-validation was used for testing the classification model with selected PCs. With each data set (all, healthy or obese), GA was run 100 times repeatedly. The PC sets that were selected the most in the repeated runs were chosen as the final result. From the results (Table 1) it can be seen that the selection from GA was quite consistent with slight variation from each run.

**Table 1:** GA selected PCs and the classification model performance (ROC)

Data for creating PCA	Result	Model _6 PCs	Model _5 PCs	Model _4 PCs	Model _3 PCs	Model _2 PCs	Model _1 PC
All	PCs selected	PC1+, PC2–, PC7+, PC11+, PC15–, PC27–	PC1+, PC2–, PC7+, PC11+, PC27–	PC1+, PC2–, PC7+, PC27–	PC1+, PC2–, PC7+	PC1+, PC7+ (or PC2–)	PC1+
	AUC (CV)	0.87	0.85	0.84	0.81	0.77	0.69
Obesity	PCs selected	PC2–, PC4–, PC14+, PC16– PC18+, PC19–	PC2–, PC4–, PC14+, PC18+ PC19–	PC2–, PC4–, PC14+, PC18+	PC2–, PC14+, PC18+	PC14+, PC18+	PC14+
	AUC (CV)	0.92	0.92	0.90	0.87	0.84	0.80
Healthy	PCs selected	PC1+,PC3+, PC5–,PC23+, PC28–,PC34+	PC1+,PC3+, PC23+,PC28– PC34+	PC1+,PC23+, PC28–,PC34+	PC1+,PC23+, PC34+	PC1+,PC34+	PC1+
	AUC (CV)	0.92	0.90	0.88	0.87	0.83	0.72

+ Positive correlation coefficient in the model

– Negative correlation coefficient in the model

The PCA constructed from the whole data set and healthy-weight subjects both created 37 principal components (PC1 to PC37) while the PCA from obese subjects created 22 components (PC1 to PC22). Table 1 lists the sets of PCs selected by GA and the cross-validated AUC produced from each prediction model built with the selected PC(s). The symbols “+” or “-” following the PC numbers indicate whether the coefficient of this PC is positive or negative in the corresponding classification model. Positive coefficient means that an increased score of this PC will increase the probability of the individual being characterised as obese. For example, PC1+ represents that the first PC created from the species abundance data will have a positive contribution to obesity with MetS.

Table 2 lists the top five species that have the highest contribution to each PC selected by GA. The symbols “+” or “-” following the species names indicate whether it has positive or negative contribution to the corresponding PC. For example, Prausnitzii- within column Comp1 represents that Prausnitzii has negative correlation with Comp1 (PC1 for Whole, PC14 for obese, and PC1 for Healthy). That suggests that increased Prausnitzii abundance will decrease the Comp1 value. As Comp1 has a positive correlation with being overweight, it can be speculated that increased Prausnitzii abundance leads to decreased likihood of being obese.

**Table 2:** Top species included in the GA selected 1, 2, 3, 4, 5 or 6 PCs produced with different data sets

Dataset for creating PCA	High contribution variables (high coefficients in the corresponding PC) included in the most selected components					
	*Comp1*	*Comp2*	*Comp3*	*Comp4*	*Comp5*	*Comp6*
*Whole (PC1, PC7, PC2, PC27, PC11, PC15)*	Prausnitzii–^a^	Gnavus+	Eutactus+^a^	Moorei–	Eggerthii–^a^	Zeae+^a^
	Eutactus–^a^	Faecis–^a^	Prausnitzii+^a^	Obeum–	Dispar–^a^	Gnavus–
	Formicigenerans–^a^	Copri+	Aerofaciens–	Lenta+^a^	Adolescentis+	Stutzeri+^a^
	Catus–^a^	Muciniphila–^a^	Catus–	Animalis–	Mucilaginosa–^a^	Bromii+^a^
	Faecis–^a^	Adolescentis–^a^	Adolescentis–`	Torques–	Aerofaciens+	Fragilis+^a^
*Obesity (PC14, PC18, PC2, PC4, PC19, PC16)*	Eutactus–^a^	Uniformis+	Dolichum–	Producta–	Caccae+^a^	Formicigenerans+^a^
	Bromii+	Catus–^a^	Lenta–	Prausnitzii+^a^	Parainfluenzae+^a^	Bromii–
	Adolescents–^a^	Dispar+	Aerofaciens+^a^	Aerofaciens–	Formicigenerans+^a^	Distasonis–
	Formicigenerans+	Faecis+	Producta–	Fragilis–	Adolescentis–	Eutactus+^a^
	Producta–^a^	Distasonis–^a^	Gnavus–	Faecis+^a^	Dispar–	Perfringens+^a^
*Healthy (PC1, PC34, PC23, PC28, PC3, PC5)*	Prausnitzii–^a^	Stutzeri–^a^	Callidus–^a^	Ovatus–	Copri+	Copri+^a^
	Eutactus–^a^	Zeae+	Moorei+	Longum+^a^	Muciniphila–^a^	Muciniphila+^a^
	Catus–^a^	Gnavus+	Formigenes+	Distasonis+^a^	Formigenes–^a^	Prausnitzii–
	Formicigenerans–^a^	Dispar+	Prausnitzii+	Fragilis–	Catus+	Formigenes+^a^
	Faecis–^a^	Lenta–^a^	Catus–^a^	Aerofaciens–	Biforme+	Eutactus+^a^

Comp1, Comp2, Comp3, Comp4, Comp5 and Comp6 represent the 6 PCs selected by GA. For experiment with whole dataset they are PC1, PC7, PC2, PC27, PC11 and PC15 respectively; for experiment with obesity sample, they are PC14, PC18, PC2, PC4, PC19 and PC16; for experiment with healthy sample, they are PC1, PC34, PC23, PC28, PC3 and PC5

^a^Species has a positive correlation with the probability of having healthy body mass

+ Positive correlation with the corresponding PC

– Negative correlation with the corresponding PC

From the results presented in Table 1 and Table 2 each of the species were analysed and categorized into two groups; positive (indicated with an asterisk (*) in Table 2) or negative correlations with the probability of having healthy body mass. The combination of having any one of the microbial species from each column can be a set of species that can have a high impact on health. For example, based on the results from the first set of the experiments which ran PCA on the whole dataset, either “Prausnitzii, Faecis, Eutactus, Lenta, Eggerthii and Zeae”, “Formicigenerans, Faecis, Eutactus, Lenta, Eggerthii and Zeae” or “Prausnitzii, Formicigenerans, Faecis, Eutactus, Lenta, Eggerthii and Zeae” can be a combination to have a potential benefit on health. On the other hand, high values for Gnavus, Catus, Moorei and Aerofaciens together are associated with high probability with of being obese.

**Fig. 2:**
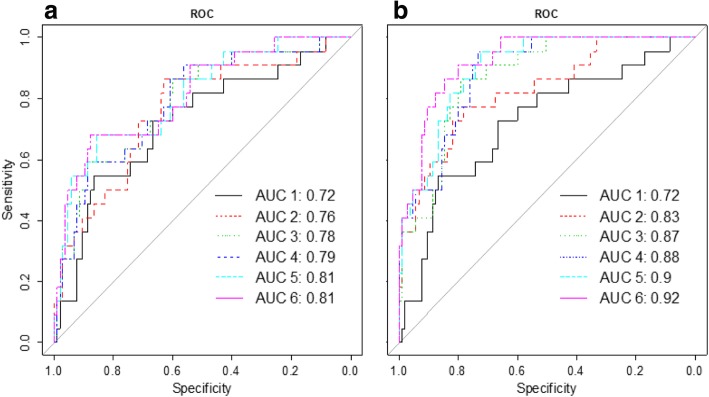
ROC produced from the top PCs of PCA and from the PCs selected by GA. **a**: 1--PC1, 2--PC1 + PC2, 3--PC1 + PC2 + PC3, 4--PC1 + PC2 + PC3 + PC4, 5-- PC1 + PC2 + PC3 + PC4 + PC5, 6-- PC1 + PC2 + PC3 + PC4 + PC5 + PC6; **b** 1--PC1, 2--PC1 + PC34, 3--PC1 + PC34 + PC23, 4--PC1 + PC23 + PC28 + PC34, 5-- PC1 + PC3 + PC23 + PC28 + PC34, 6--PC1 + PC3 + PC5 + PC23 + PC28 + PC34

A final classification model was built with each set of PCs selected by GA or first 1 to 6 PCs (which explain the most variance of the data) from the PCA. Again, the PCs were calculated from the whole dataset, healthy-weight dataset or obese dataset. The AUCs produced from the GA-selected PCs were quite obviously higher than the ones from the top PCs of PCA. Figure 2 shows the ROCs created from the models built with the selected PCs and the first PCs of the PCA. The PCs in the graph were calculated with the healthy-weight dataset (when compared with the result from the PCs calculated from whole dataset and obese dataset, the first PCs from the healthy data produced the highest AUC values).

### GA models to select sets of species for classification

To compare the results from the model that combined PCA and GA and from the model where GA was applied directly for selection of the combination of the bacterial species, GA was implemented in conjunction with logistic regression using the species abundance directly as the input for classification.

**Fig. 3:**
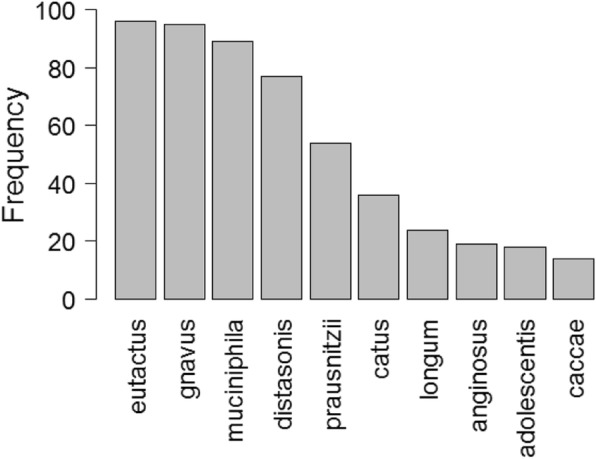
Frequencies of the species selected from the multiple runs of GA. The GA was run with logistic regression as the classification model and species abundance as the input. GA was used to select the combination of the species for classification of obese individuals from the health group. The number on top of each bar is how many times out of 100 GA runs the corresponding species was selected

For experiments, the number of species (number of input variables for logistic regression) was restricted to maximum six, which was the same as the maximum number of PCs used in the earlier experiments. Table 3 shows the combinations of the bacterial species selected by 100 repeated runs of GA, which achieved the highest AUC values. It can be seen that some of the species were commonly selected in different sets of the selections. The selection frequency of each species from the 100 repeated GA runs was calculated and a frequency chart showing the top 10 most selected species was drawn in Fig. 3. Eutactus and Gnavus appeared in the final selection of almost every run of the GA (96 out of 100 runs and 95 out of 100 runs). Muciniphila, Distasonis and Prausnitzii were also selected frequently (> 50% frequency) in the repeated GA runs. These highly selected bacterial species appeared to have relatively high contribution to the selected PCs shown in the previous section.

**Table 3:** Sets of species selected by GA using the species abundance as the input variables of logistic regression models

GA Selected Species						AUC
Adolescentis	Catus	Eutactus	Gnavus	Muciniphila	Prausnitzii	0.87
Adolescentis	Distasonis	Eutactus	Gnavus	Muciniphila	Prausnitzii	0.87
Aerofaciens	Distasonis	Eutactus	Gnavus	Longum	Muciniphila	0.88
Anginosus	Distasonis	Eutactus	Gnavus	Muciniphila	Prausnitzii	0.87
Catus	Distasonis	Eutactus	Gnavus	Muciniphila	Prausnitzii	0.88
Catus	Eutactus	Gnavus	Longum	Muciniphila	Prausnitzii	0.86
Distasonis	Eutactus	Gnavus	Longum	Muciniphila	Prausnitzii	0.88

GA Selected Species lists the set of species selected by GA, each row one set. AUC is the area under the ROC curve produced by the corresponding logistic regression model with the selected set of species. The result was cross validated with the same cross validation set up as the earlier experiments

## Discussion

In this study, a computational method that combines PCA and GA has been proposed to produce accurate prediction result and to find sets of features (variables) that contribute the most to the prediction models. The model was applied to identify sets of bacterial species associated with high body mass. Due to the high correlation between many species of the gut bacteria, constructing PCA before the GA can improve the efficacy of GA for selecting multiple sets of microbial species associated with obesity and MetS. The result from this study showed that the prediction models built with the PCs selected by GA produced much higher AUC values than the models built with the top PCs that explained the greatest proportion of the variance in the sample. The results were also compared with those produced from the GA selected models with bacterial species abundance values as the input directly, and it showed its advantages.

In the microbiome study, the results produced from the described method depends on the accuracy of the sequencing analysis. The microbial species identified here was based on the sample of 22 obese subjects and 105 healthy-weight subjects. Assuming this result was validated in multiple datasets with bigger sample sizes, the results from Table 1 and Table 2 can suggest a few combinations of microbial species groups that are beneficial to health. Some of the species in the combinations can be replaced by equivalent alternative species that are suggested by the algorithm, which gives flexibility for further intervention. As described in the previous sections, the bacterial species detected can be different when applying different sequencing analysis and taxonomy classifications. To validate the findings from this study, the presented algorithm should be run with the outcomes from metagenomics sequencing and with other sequencing analysis pipelines. Different reference databases (e.g. NCBI) can also be used for taxonomy classification of the OTUs identified.

## Conclusion

This study demonstrated the value of applying GA for selection of subsets of PCs from PCA to improve the performance of prediction models. The features included in the PCs that were selected by GA can be combined with flexibility for potential clinical applications. With the flexible options of combining the features included in the PCs selected by the GA, different interventions can be recommended for different patients, which contributes to the practice of personalised medicine. The proposed algorithm was designed in a general way and was tested in a study comparing obese individuals with MetS and healthy-weight subjects. It can be applied for any other classification or biomarker identification study. The model takes into account correlations of the variables (bacteria species in this study) and the advantages of GA for feature selection. It overcomes the limitations of the ways in which PCAs are currently used for prediction modelling. The algorithm can be useful for many biological studies where high dimensional data are collected with strongly correlated variables.

## Data Availability

Data are available upon request from the Menzies Health Institute Queensland for researchers who meet the criteria for access to confidential data.

## Abbreviations

AUC: Area under the ROC
BMI: Body mass index
GA: Genetic algorithm
MetS: Obesity with metabolic syndromes
OTU: Operational taxonomic unit
PC: Principal component
PCA: Principal compoment analysis
ROC: Receiver operator characteristic curve
